# PlantSize Offers an Affordable, Non-destructive Method to Measure Plant Size and Color *in Vitro*

**DOI:** 10.3389/fpls.2018.00219

**Published:** 2018-02-22

**Authors:** Dóra Faragó, László Sass, Ildikó Valkai, Norbert Andrási, László Szabados

**Affiliations:** Institute of Plant Biology, Biological Research Centre, Szeged, Hungary

**Keywords:** *Arabidopsis thaliana*, PlantSize, color imaging, rosette size, chlorophyll content, anthocyanin content, heat shock factor A4A, stress responses

## Abstract

Plant size, shape and color are important parameters of plants, which have traditionally been measured by destructive and time-consuming methods. Non-destructive image analysis is an increasingly popular technology to characterize plant development in time. High throughput automatic phenotyping platforms can simultaneously analyze multiple morphological and physiological parameters of hundreds or thousands of plants. Such platforms are, however, expensive and are not affordable for many laboratories. Moreover, determination of basic parameters is sufficient for most studies. Here we describe a non-invasive method, which simultaneously measures basic morphological and physiological parameters of *in vitro* cultured plants. Changes of plant size, shape and color is monitored by repeated photography with a commercial digital camera using neutral white background. Images are analyzed with the MatLab-based computer application PlantSize, which simultaneously calculates several parameters including rosette size, convex area, convex ratio, chlorophyll and anthocyanin contents of all plants identified on the image. Numerical data are exported in MS Excel-compatible format. Subsequent data processing provides information on growth rates, chlorophyll and anthocyanin contents. Proof-of-concept validation of the imaging technology was demonstrated by revealing small but significant differences between wild type and transgenic Arabidopsis plants overexpressing the *HSFA4A* transcription factor or the *hsfa4a* knockout mutant, subjected to different stress conditions. While *HSFA4A* overexpression was associated with better growth, higher chlorophyll and lower anthocyanin content in saline conditions, the knockout *hsfa4a* mutant showed hypersensitivity to various stresses. Morphological differences were revealed by comparing rosette size, shape and color of wild type plants with phytochrome B (*phyB-9*) mutant. While the technology was developed with Arabidopsis plants, it is suitable to characterize plants of other species including crops, in a simple, affordable and fast way. PlantSize is publicly available (http://www.brc.hu/pub/psize/index.html).

## Introduction

Plant phenotype is determined by the genetic background and environmental conditions. Interaction of the genotype and environmental factors influences plant growth and development, physiological and molecular traits. Characterization of phenotypes therefore requires precise description and monitoring of multiple structural and physiological traits. Standard methods are available to measure plant size, shape, and structure at different levels, and get information about numerous physiological and molecular characters. While classical methods are generally precise and reliable, they usually destroy the plant, and provide information at the endpoint of the experiment. Besides, standard physiological techniques often require numerous analytical steps and measurements, making large-scale analysis difficult or impossible. Analysis of large number of plants is therefore a time-consuming and error-prone procedure.

To circumvent such limitations, non-destructive methods have been developed to analyze different morphological and physiological parameters. Such methods are usually based on imaging technologies, which allow serial measurements, and simultaneous detection of several morphological and physiological parameters (Furbank and Tester, 2011; Dhondt et al., 2013; Rungrat et al., 2016). Analysis of color photographs by computer applications is a key component of such non-destructive measurements, which generates numerical data from the digital images allowing the application of complex statistical evaluation (Spalding and Miller, 2013; Sozzani et al., 2014). Numerous softwares have been developed to analyze different parameters of model and crop plants^1^. Measurements include size and structure of different organs such leaves and shoots (Bylesjo et al., 2008; Weight et al., 2008; Jansen et al., 2009; Dhondt et al., 2014; Apelt et al., 2015; Tome et al., 2017), grass stalk structure (Heckwolf et al., 2015), seedling size and phenotype (Walter et al., 2007), hypocotyl (Wang et al., 2009), root architecture (Brooks et al., 2010). Applications are available to characterize certain physiological parameters such as chlorophyll content (Majer et al., 2010; Liang et al., 2017) or chlorophyll fluorescence (Jansen et al., 2009; Awlia et al., 2016; Rungrat et al., 2016). To handle large number of plants, automatic phenotyping platforms have been developed, which are able to acquire large sets of data, characterizing plant growth and physiological status in a non-destructive manner (Dhondt et al., 2013; Feher-Juhasz et al., 2014; Junker et al., 2014; Mutka and Bart, 2014; Awlia et al., 2016). While phenotyping platforms have usually been developed for crop plants, such technologies have also been adapted for the analysis of smaller model plants such as *Arabidopsis thaliana* (Arvidsson et al., 2011; Dhondt et al., 2014; Apelt et al., 2015; Awlia et al., 2016). Complex phenotyping systems are available from several commercial companies such as LemnaTec^2^, Photon Systems Instruments (PSI^3^) or WIWAM^4^. Such automatic phenotyping, however, rely on sophisticated and expensive equipment, and personnel experienced in image analysis, not available for most research laboratories.

*In vitro* conditions offer homogeneous, well-controlled environment to study plant growth and development. Non-destructive image analysis can be adapted to *in vitro* systems to measure growth and physiological parameters of different plants, including the most commonly used model, *Arabidopsis thaliana*. Rosette of young Arabidopsis can be considered as a two-dimensional structure, which is proportional to the biomass of the plant. 2D digital imaging can therefore be used to record rosette parameters and monitor growth under variable conditions. Images can be evaluated by open-source applications such as ImageJ (Schindelin et al., 2015) and Fiji (Schindelin et al., 2012), or by particular computer applications, which have been developed to analyze plant images and get quantitative information on plant size and development (De Vylder et al., 2012; Vanhaeren et al., 2015). Recently an automatic custom-made *in vitro* growth imaging system (IGIS) has been developed, which can perform multiple measurements and is suitable to monitor plant growth in sterile cultures (Dhondt et al., 2014).

Photosynthetic capacity in plants is closely related to chlorophyll content in leaves, which is therefore an important physiological indicator. Chlorophyll content has traditionally been determined by chemical extraction and spectrophotometric measurements (Lichtenthaler and Buschmann, 2001). Non-destructive methods has already been developed which permit the estimation of chlorophyll contents without sacrificing leaves or whole plants. SPAD chlorophyll meter readings are based on transmittance of red and infrared light through the leaves (Markwell et al., 1995; Adamsen et al., 1999; Uddling et al., 2007; Ling et al., 2011). Spectral reflectance is another valuable tool to determine chlorophyll content in plants (Gitelson et al., 2003). Analysis of RGB color components of digital photographs was employed to determine chlorophyll contents in different plants and environmental conditions (Adamsen et al., 1999; Majer et al., 2010; Riccardi et al., 2014; Awlia et al., 2016; Liang et al., 2017). While non-invasive optical methods are useful to estimate leaf chlorophyll contents, calibration for different species and leaf types is essential to get reliable results (Richardson et al., 2002). Hue values of leaf color were previously shown to correlate with chlorophyll content and are suitable to estimate photochemical yield of photosystem II (Majer et al., 2010; Sass et al., 2012; Minervini et al., 2017).

While most imaging applications are useful to get reliable data on a particular parameter, an affordable imaging software with complex analytical capability is still missing. Here we describe a novel imaging system, which is based on the analytical tool PlantSize, and is suitable for fast and reproducible analysis of important morphological parameters and color components. Several examples illustrate the utility of the software, which include the characterization of transgenic Arabidopsis lines overexpressing the heat shock factor A4A and the insertion mutant *hsfa4a*. The software is freely available with documentation and detailed user guide.

## Materials and Methods

### Plant Growth and Development

If otherwise not stated, Arabidopsis plants were grown on agar-solidified half strength MS culture medium containing 0,5% sucrose (1/2MS) as described (Szabados et al., 2002). For growth assays, seeds were germinated on 1/2MS medium and 5-days-old seedlings were transferred to fresh culture media in square Petri dishes, arranged in a matrix. Plants were grown in controlled growth chambers, under 120 mE illumination using 12/12 h light/dark cycle.

### Stress Treatments

Growth assays were performed by transferring 5-days-old seedlings to agar-solidified culture media supplemented by 100 mM NaCl or 0.2 μM paraquat. High stress treatments were made on *in vitro*-grown 2-weeks-old plants by transferring them to culture media solidified by 0,4% agar and supplemented with different concentrations of NaCl (150, 200 mM), CdCl_2_ (0,1 mM), hydrogen peroxide (3 mM), sprayed with paraquat (1, 3, 5 μM) or hydrogen peroxide (10, 20 mM). Plants were cultured in these conditions for 3 days and images were taken at daily intervals.

### Determination of Chlorophyll and Anthocyanin Contents

For chlorophyll determination, plants with different tones of green were collected, and their fresh weights were determined individually. Plants were extracted with 95% ethanol at 4°C, overnight. Chlorophyll content was determined as described (Lichtenthaler and Buschmann, 2001), measuring absorbances at 470, 648, and 664 nm using Multiskan G0 spectrophotometer (Thermo Scientific). Absorbance was measured between OD 0.3 and 0.8. Chlorophyll concentrations were calculated with equations as reported by Lichtenthaler and Buschmann (2001).

Chlorophyll a (μg/mL) = 13.36 A664.1 – 5,19 A648.6
Chlorophyll b (μg/mL) = 27.43 A648.6 – 8.12 A664.1
Carotenoids (μg/mL) = (1000 A470 – 2.13 Chl a – 97.64 Chl b)/209
Total chlorophyll (μg/mL) = Chl a + Chl b
Chlorophyll concentrations were calculated based on fresh weight (mg FW).

For anthocyanin determination fresh weights of plants were measured, which were frozen in liquid nitrogen and grinded. Plant material was resuspended in distilled water (200 μL/plant) and centrifuged in microcentrifuge at 13000 rpm, 4°C, for 10 min. Supernatant was removed and total anthocyanin content was determined by the pH-differential method as described (Giusti and Wrolstad, 2001). Two 100 μL samples were separated into two microcentrifuge tubes and pH were adjusted to pH1.0 and pH4.5 by adding 400 μL 0.025M potassium chloride buffer (pH 1.0) and 0.4M sodium acetate buffer (pH 4.5), respectively, and incubated on ice for 15 min. Absorbance of both samples were determined at 520 and 700 nm, using Multiskan G0 spectrophotometer (Thermo Scientific). Difference of absorbance was calculated as follows: Adiff = (A520–A700) pH1.0 – (A520–A700) pH4.5. Concentration of monomeric anthocyanins were calculated using the following formula: *Adiff*x83,5 (mg/liter). Anthocyanin concentrations were normalized to fresh weight (mg FW).

### Calibration of the System

To calibrate the imaging system for plant size determination, 10–12-days-old Arabidopsis plants were photographed and subsequently carefully removed from the culture medium, to measure fresh and dry weights individually. Dry weights were determined after dehydration in 80°C oven for 24 h. For calibration, rosette sizes (pixels) and fresh and/or dry weights of 250 individual plants were compared.

To calibrate the system for chlorophyll or anthocyanin determination, 4-weeks-old *in vitro* grown plants were subjected to different stress conditions (0,1 mM CdCl_2_, 3 mM hydrogen peroxide, 0.2 or 0.5 M saccharose. Plants with different tones of green or purple coloration were photographed and collected individually for chlorophyll or anthocyanin determination. Chlorophyll and/or anthocyanin content of individual plants were measured and compared to HUE values of the same plants, photographed before sample collection. For calibration of chlorophyll and anthocyanine content, three hundred plants were used which gave satisfactory results.

Calibration is recommended before using the PlantSize software in a new environment or experimental setup. Culture conditions might influence morphological parameters such as leaf thickness, plant shape. Imaging with different light sources, conditions determine color and subsequently influence HUE values, which are employed to quantify chlorophyll and anthocyanin contents. Therefore, such parameters will have to be calibrated by the user for the plants to be analyzed and imaging conditions used. Calibration with two to three hundred plantlets seem to give satisfactory results to establish correlation between the parameters measured.

### Image Capture

Plant growth was monitored in time by taking photographs at regular intervals (usually every 3 days) over a 2 weeks assay period. Color images were taken by photographing the plates with white, transmission illumination of a transilluminator (Stratagene) or on white surface using homogeneous upper incandescent illumination. When growth over 10–14 days was monitored, closed Petri dishes were photographed upside down on transilluminator, which eliminates problems of humidification of the lids. For short time monitoring (up to 3 days) Petri dishes were photographed without lid from above, using a white, homogeneous surface. In our experiments Canon PowerShot SX20 digital camera was used without any filter, but any other digital camera able to take 3000 × 4000 pixel images can be employed. In a typical experiment the following settings were used: ISO: 100, shutter speed: 1/30 s, Aperture: F/5.6, Manual focus, Exposure mode: macro. Image resolution was adjusted to 3000x4000 pixels. Images were saved in jpeg format. Depending on the experimental conditions, light source and intensity, settings should be optimized before large-scale measurements.

### Image Analysis

The PlantSize software was developed for multiple image analysis using MATLABs (version 2016b) with the Image Processing Toolbox^TM^ (The MathWorks Inc., Natick, MA, United States)^5^. Image processing with PlantSize is described in Supplementary Data 1. System requirements are the following:

– 4 GB RAM (recommended)
– Windows 64 bit (Windows7 or Wimndows10)
– Screen with 1920 × 1080 pixel resolution.
– MATLAB Runtime version R2016b installed: http://www. mathworks.com/products/compiler/mcr/index.html. Online help is available: http://www.mathworks.com/help
– Installed PlantSize.exe software^6^. Potential users are encouraged to check for updates and consult with the developer.

Steps of image analysis are the following. Detailed description of the image analysis is described in Supplementary Data 2.

(1) Start PlantSize. A welcome window appear, Click “OK.”
(2) Import image file into PlantSize software: File Menu > LOAD function, select for the desired image file (.jpg), load the image into the Main menu (Supplementary Figure S2).
(3) Project’s name: You can set the name of the project (optional). If not, the exported file will have the name of the image.
(4) Date: you can set the date (optional).
(5) Name: you can define the name of the analysis (optional).
(6) Define the number of rows and columns which generates the matrix for analysis: Divisor X (column), Divisor Y (row).
(7) Define names of rows: it is recommended to arrange a plant genotype in the same row of the matrix. Enter the corresponding names to the Rows.
(8) Press Select the area for analysis. The system automatically creates an evenly distributed grid for analysis. Alternatively you can define the number of each row and column. Logically define the grid according to the matrix of your plants.
(9) Color space: The Red-Green-Blue (RGB) color space is converted to HUE, Saturation and Value color space (HSV) using the Image Processing Toolbox^TM^. (The MathWorks Inc., Natick, MA, United States) (Sass et al., 2012).
(10) Segmentation: Background is removed and objects (plants) are defined with the saturation and value sliders, which set the sensitivity of the system. “Green” slider defines saturation value, “Gray” slider will set intensity in grayscale. The software will recognize plants according to the green and gray settings. Define settings at the beginning of a set of measurement, which will automatically applied to each image.
(11) Request of Interest (ROI) function (Magnify window): if necessary a cell can be enlarged and the polygone around the rosette can be defined. Close the enlarged cell by double click.
(12) Calculation: press “OK” button. The adjusted mask is accepted and parameters are calculated.
(13) If you press “Show values” button, the numerical values associated with each grid (plant) will appear.
(14) Save data: File Menu > Save. Data will be saved in.xls format, exported to the same folder with the image.
(15) If multiple images are analyzed, do not close PlantSize, but open the next image file. Each dataset will be exported to the same file to different sheets.
(16) Exported numerical data can be further analyzed by MS Excel or other statistical applications.

### Features

Images of up to 36 plants were analyzed simultaneously in our experimental conditions. Number of plants depend on the size of the plants, the imaged area, the capacity of separation individual plants without cropping of leaves. When necessary, individual plants can de dissected manually, but that is a rather time-consuming process.

Numerical data are exported to a MS Excel formatted file. PlantSize generates data on the following characters: rosette size (Pixel Area, Weight), chlorophyll content (μg Chl./pixel), anthocyanin content (μg Anth./pixel), Convex area (pixels), and Convex ratio (%). Convex area shows how large is the area within convex hull, convex ratio compares convex area and rosette size. These calculations provide data about the shape of the leaves and rosette (Vanhaeren et al., 2015). Subsequent data processing can be performed in MS Excel. Saved data can be imported or copied into MS Excel or other statistical software to perform statistical analysis, what can include calculation of averages, standard deviation, standard error, significance, etc. We have applied analysis of variance (one way ANOVA) with Tukey test of significance for each trait (*p*-value < 0.05).

## Results

### The Image Analysis System

**FIGURE 1:**
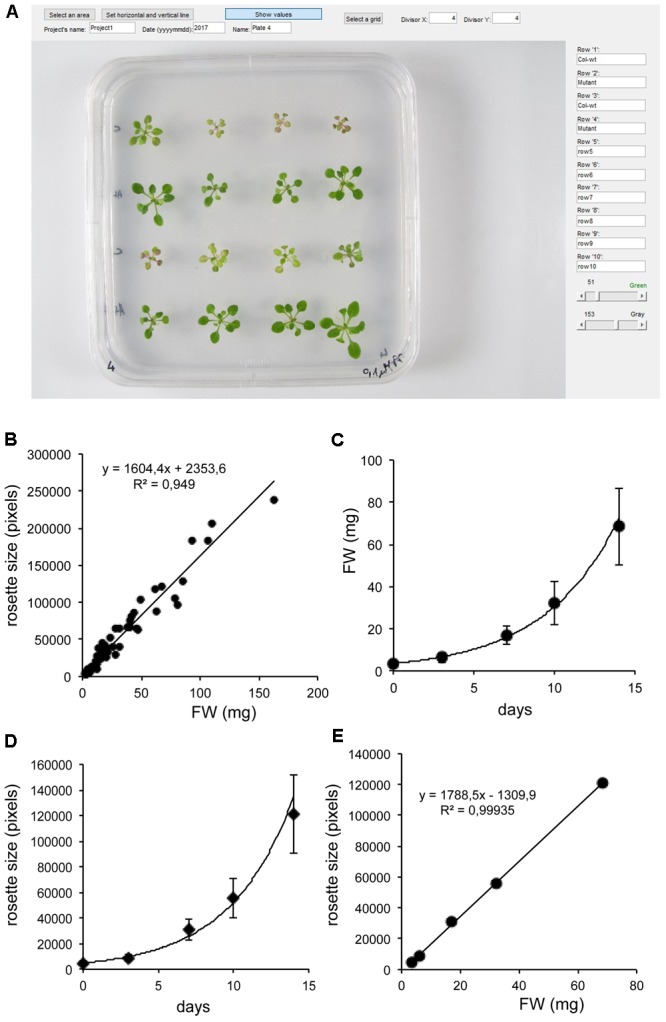
Use of PlantSize to measure rosette sizes of Arabidopsis plants. **(A)** Dialog box of PlantSize with imported images of young wild type and mutant Arabidopsis plants. **(B)** Linear correlation of fresh weights and rosette sizes of individual Arabidopsis plants, grown on standard culture medium. Note high level of correlation between rosette sizes (shown in pixel numbers) and fresh weights (FW) of individual plants. **(C)** Change of average fresh weights of wild type Arabidopsis plants in 14 days growth period. **(D)** Change of average rosette sizes of wild type Arabidopsis plants in 14 days growth period, as determined by PlantSize. **(E)** Linear correlation of average fresh weights and average rosette sizes displayed on **C,D**.

To facilitate the rapid and easy evaluation of plant growth, a Matlab-based image analysis application was created and optimized to analyze basic characteristics of Arabidopsis plants, cultured in *in vitro*. Protocols for quantitative analysis of rosette size, shape and color were developed, which allowed the simultaneous determination of growth rates, convex areas and percentages as well as measurement of chlorophyll and anthocyanin contents. For image analysis, plants were grown in square petri dishes on agar-solidified culture medium, arranged in a matrix (up to 6 × 6 = 36 plants / Petri dish were tested, Supplementary Figure S1). Images were imported to PlantSize and were processed to generate numerical data of various parameters describing size, shape and color of the plants identified on the image (**Figure 1A** and Supplementary Figure S2). In a typical time-series experiment consisting of 4 treatments and 6 Petri dishes/treatment, 24 Petri dishes were photographed in one time point. With 5 time points, 120 photographs were generated having 4320 plants images, which could easily be processed by PlantSize, simultaneously generating numerical data of several important features.

### Analysis of Rosette Sizes

Image analysis permits the non-destructive determination of plant sizes and estimation of growth rates. To calibrate our image analysis system, wild type Arabidopsis plants were grown on standard culture medium and plants of different sizes were photographed and analyzed (Supplementary Figures S1, S2). Rosette sizes of individual plants were determined by PlantSize and compared to fresh weights. Linear correlation between these parameters could be observed (*R*^2^= 0.95, **Figure 1B**), confirming that rosette size measured in pixels is a reliable feature to characterize plant growth over time. In this particular experiment 1 mg difference in fresh weight corresponded to approximately 1600 pixels in rosette sizes (at 3000 × 4000 pixels image size). Non-destructive imaging allows monitoring plant growth over time. We have grown plants for 14 days, comparing changes in fresh weights (FW) and rosette sizes. Logarithmic growth of FW and pixel numbers could be established, showing high degree of correlation when average values were compared (*R*^2^= 0.99) (**Figures 1C–E**).

To validate our system with other established methods, rosette sizes were measured with PlantSize and Image J^7^, a free software, used frequently to measure sizes of digital images (Schindelin et al., 2015). High degree of correlation was found in leaf areas determined with PlantSize and ImageJ (Supplementary Figure S3), showing that data obtained by these applications are comparable. PlantSize, however, can perform simultaneous analysis of numerous plants (up to 36 plants were tested in the present version), measuring not only size but other parameters as well, which is a clear advantage for high throughput analysis.

**FIGURE 2:**
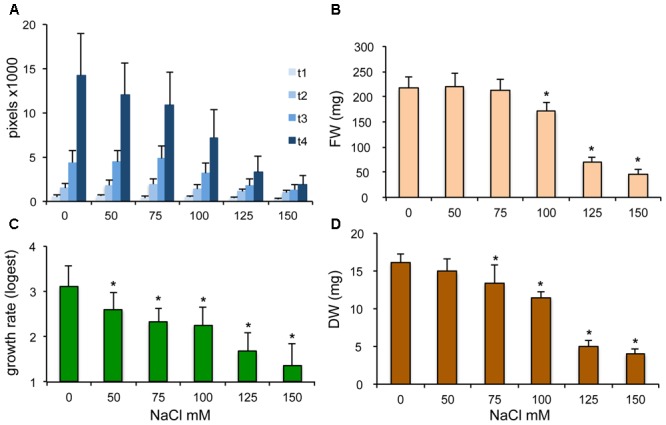
Repression of Arabidopsis growth by increasing concentrations of salt. Five days-old seedlings were transferred to media supplemented by different concentrations of NaCl. Growth was monitored either by rosette imaging or weight measurements. **(A)** Rosette sizes of Arabidopsis plants determined by imaging and PlantSize analysis. **(B)** Average growth rates of plants calculated by the “Logest” function of Excel. **(C,D)** Average fresh weights (FW) and dry weights (DW) of plants grown on saline media. Bars on diagrams indicate standard deviation, ^∗^shows significant differences to control tested by one-way ANOVA (*p* < 0.05).

Imaging systems are often used to monitor plant growth in different environmental conditions. To quantitate the effect of salinity on Arabidopsis, plant sizes were determined periodically on culture media supplemented by 0 to 150 mM NaCl. Rosette sizes, growth rates, fresh and dry weights were compared to characterize growth-reducing effect of salt (**Figure 2**). While salt had clear inhibitory effect on rosette growth, considerable variation could be observed in all NaCl concentrations tested (Supplementary Figure S4). Rosette sizes and growth rates were significantly reduced even by mild salt stress, while fresh and dry weights were significantly affected only by 100 mM or higher concentations of NaCl. These data suggest, that leaf area, calculated by PlantSize, is a more sensitive and reliable parameter than fresh or dry weights, and is well suitable to monitor the detrimental effects of adverse conditions such as salinity, in non-destructive way.

### Estimation of Chlorophyll and Anthocyanin Content

**FIGURE 3:**
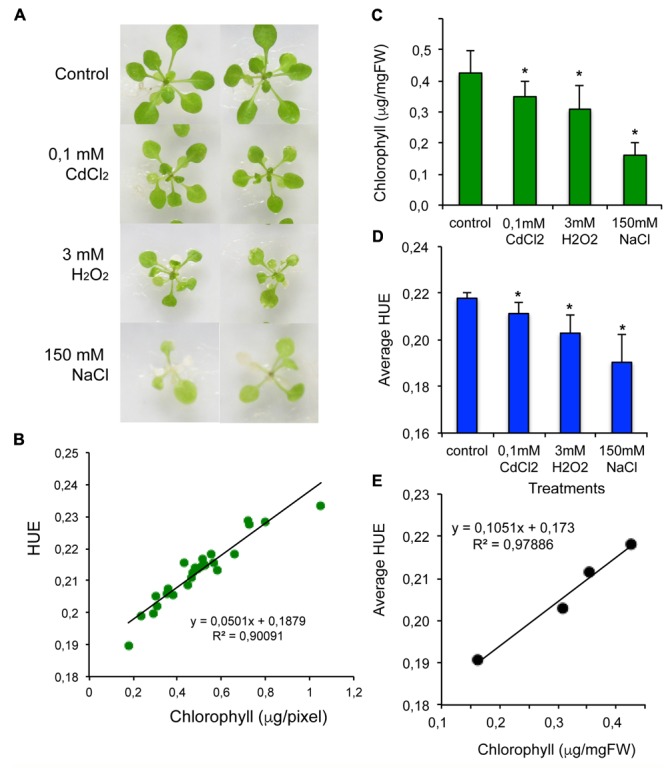
Correlation of chlorophyll contents with Hue values. Fourteen days-old Arabidopsis plants treated by different stresses: 0,1 mM CdCl_2_, 3 mM H_2_O_2_, 150 mM NaCl, known to affect chlorophyll content. Plants were photographed after 3 days, and images were analyzed by PlantSize. **(A)** Images of treated plants. **(B)** Linear correlation of Hue values and chlorophyll contents of individual Arabidopsis plants (pixel numbers were used according to **Figure 1B**). **(C,D)** Average chlorophyll contents and HUE values of treated plants. **(E)** Correlation of average chlorophyll contents and average HUE values. Bars on diagrams show standard deviation, ^∗^Indicates significant differences to control tested by one-way ANOVA (*p* < 0.05).

Image-based phenotypic analysis offers a non-destructive technology to estimate the amount of colored compounds in living tissues, such as chlorophyll and anthocyanin, by detecting differences in color intensity and spectrum. To calibrate our system for color analysis, *in vitro*-grown Arabidopsis plants were subjected to different treatments, known to degrade chlorophyll by promoting the formation of reactive oxygen species (CdCl_2_, H_2_O_2_, NaCl, **Figure 3A**). Leaf HUE of color images were previously used to estimate chlorophyll content, and shades of green were shown to correspond to HUE values between 0.15 and 0.25 (Majer et al., 2010). Chlorophyll contents were determined in individual plants and compared to HUE degrees of color images taken previously of the same plants. When HUE values were plotted to chlorophyll contents expressed as μg/mgFW, a saturating exponential curve fitted best to the correlation (not shown). Similar, non-linear relationship of leaf Hue values and leaf chlorophyll content was observed previously, reporting non-linearity at low chlorophyll concentrations (Majer et al., 2010). Linear correlation could, however, be observed in a concentration range (from 0.2 to 1.0 μgChl/pixel), when HUE values were plotted to chlorophyll contents based on pixel numbers of rosette sizes (**Figure 3B**). Salt, oxidative and heavy metal stresses were found to reduce chlorophyll content and shift HUE of leaf color (**Figures 3C,D** and Supplementary Figure S5). Reduced chlorophyll correlated well with lower HUE, when average values of 20 plants were compared (**Figure 3E**). These data show, that reliable estimation of the chlorophyll content can be obtained in Arabidopsis plants by non-destructive imaging using the PlantSize software.

**FIGURE 4:**
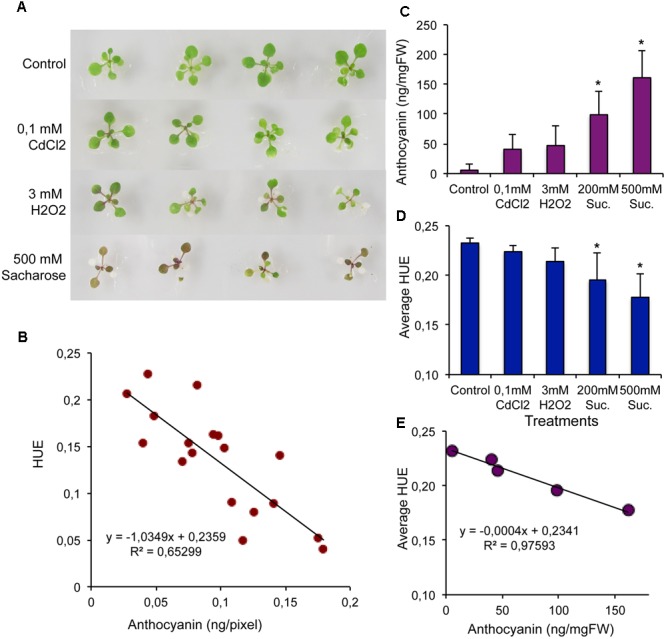
Estimation of anthocyanin accumulation in Arabidopsis plants. Fourteen days-old Arabidopsis plants were treated by 0,1 mM CdCl_2_, 3 mM H_2_O_2_, 200 mM, and 500 mM sucrose, known to stimulate anthocyanin accumulation. Plants were photographed after 3 days and images were analyzed by PlantSize. **(A)** Images of plants with different anthocyanin content. **(B)** Reverse correlation of Anthocyanin contents and HUE values of individual Arabidopsis plants (pixel numbers were used according to **Figure 1B**). **(C,D)** Average anthocyanin contents and average HUE values of treated plants. **(E)** Reverse correlation of average anthocyanin contents and HUE values. Bars on diagrams indicate standard deviation, ^∗^shows significant differences to control tested by one-way ANOVA (*p* < 0.05).

Anthocyanin accumulation is a characteristic defense reaction of higher plants, which commonly takes place in response to extreme environmental conditions (Tanaka et al., 2008). To calibrate the imaging system, anthocyanin levels and Hue values of color images were compared in 4-weeks-old Arabidopsis plants, subjected to treatments, known to induce anthocyanin accumulation (heavy metal, oxidative stress, high sugar, etc. **Figure 4A**). Reverse correlation was observed between leaf HUE values and anthocyanin contents, although variability between individual plants was high (**Figure 4B**). While anthocyanin content increased with stress, average HUE values of these plants were reduced (**Figures 4C,D**). Non-treated control plants had a narrow green spectrum corresponding to chlorophyll content, while anthocyanin accumulation resulted in a shift from turquoise to red spectrum, and correlated with lower HUE values (-0.02 – 0.24, Supplementary Figure S5). Inverse correlation was obvious when averages of anthocyanin content and HUE were plotted (*R*^2^ = 0.98, **Figure 4E**). These data show, that anthocyanin content can be estimated in living plants based on spectral changes in leaf color. Our data show, that shifts in leaf HUE values can provide a reliable estimation of changes in chlorophyll and anthocyanin contents. Very narrow range of HUE was found to correspond to changes in chlorophyll and anthocyanin contents. Reverse linear correlations could be established between HUE and chlorophyll or anthocyanin contents, permitting the use of the simple linear model by the PlantSize tool. The PlantSize-based method offers a simple analytical tool to reveal tendencies in size and color associated with different environmental effects.

### Stress Responses of the HSFA4A Overexpression Lines and the *hsfa4a* Mutant

**FIGURE 5:**
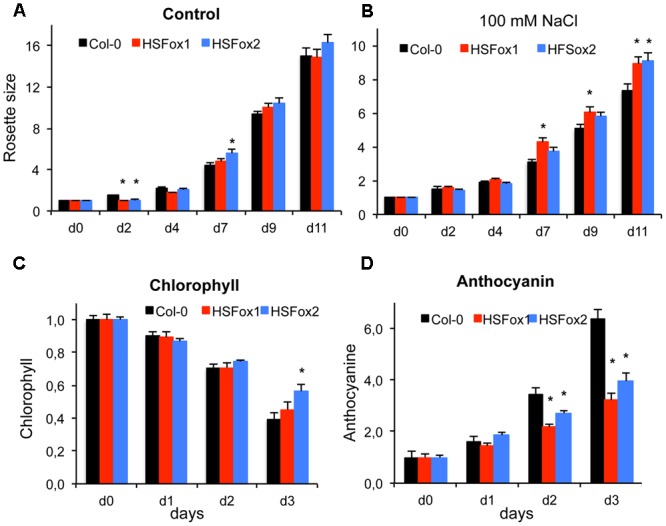
Heat shock factor A4A (HSFA4A) modulates stress tolerance. **(A)** 5-days-old *HSFA4A* overexpressing seedlings (lines HSFox1, HSFox2) and wild type plants were transferred to culture medium supplemented by 100 mM NaCl. Growth was monitored by periodic imaging and evaluated by PlantSize. **(A)** Relative rosette sizes of plants grown on standard culture medium (1 corresponds to pixel No. on day 0). **(B)** Plant growth on saline medium. **(C,D)** 14-days-old plants were transferred to medium containing 150 mM NaCl and photographed at daily intervals. Changes in chlorophyll **(C)** and anthocyanin **(D)** contents were calculated from shifts in HUE values of the color images. Relative values are shown, where 1 corresponds to values of non-treated plants (day 0). Error bars indicate standard deviation, ^∗^shows significant differences to control tested by one-way ANOVA (*p* < 0.05).

The heat shock factor A4A (HSFA4A) was previously implicated in responses to salt and oxidative stress, showing that regulated overexpression of *HSFA4A* could confer salt tolerance to Arabidopsis plants (Perez-Salamo et al., 2014). To test the utility of our system, rosette growth, chlorophyll and anthocyanin accumulation of transgenic lines with constitutive overexpression of *HSFA4A* transcription factor, as well as a *hsfa4a* knockout mutant were compared to wild type plants subjected to different stresses. Analysis of rosette sizes revealed that growth of wild type and *HSFA4A* overexpressing plants were similar in standard growth conditions, while HSFox plants were more tolerant to salt (**Figures 5A,B**). To investigate changes in chlorophyll and anthocyanin contents of these lines, 2-weeks-old, *in vitro* grown plants were transferred to media containing 150 mM NaCl, and photographed in three consecutive days. Evaluation of color images by PlantSize revealed shifts in HUE values, suggesting changes in chlorophyll and anthocyanin contents. Salt stress reduced chlorophyll content in all plants, which was less dramatic in the HSFox2 plants when compared to wild type (Col-0). Anthocyanin accumulation of salt-treated plants was 20–50% lower in *HSFA4A* overexpressing plants than in Col-0 wild type ones (**Figures 5C,D**).

When *hsfa4a* mutant and corresponding wild type plants were treated by different stresses (150 mM NaCl, 3 mM H_2_O_2_, 0,1 mM CdCl_2_), rosette sizes of the mutant were more reduced by salt and cadmium. While chlorophyll levels did not differ significantly, anthocyanin accumulation was more pronounced in the mutant upon these treatments (Supplementary Figure S6). Differences in plant size and color could be recorded by our image analysis system, suggesting stress hypersensitivity of the *hsfa4a* mutant.

### Analysis of Rosette Shape of *phyB-9* Mutant and Wild Type Plants

**FIGURE 6:**
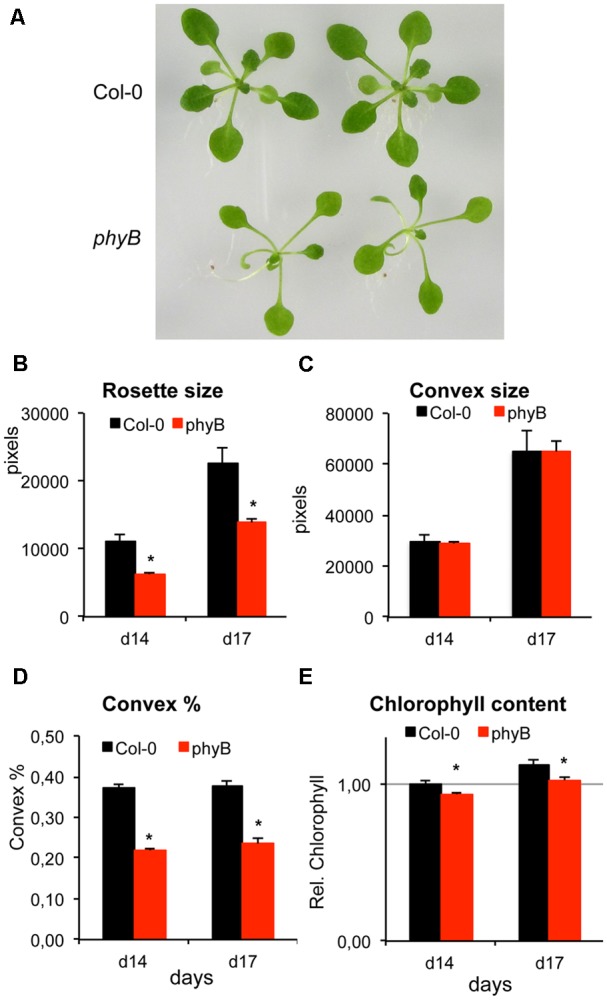
Phenotype of *phyB-9* mutant and wild type plants. Wild type (Col-0) and phytochrome B mutant (*phyB-9*) plants were cultured on standard culture medium and photographed 14 and 17 days after germination. **(A)** 17 days-old Col-0 and *phyB-9* plants. Note differences in rosette shape and color. **(B–E)** Comparison of rosette size, shape and color of 14- and 17-days-old plants. **(B)** Rosette sizes in pixels. **(C)** Convex size in pixels. **(D)** Convex % of wild type and *phyB* plants. **(E)** Chlorophyll content determined from HUE. Relative values are shown, which were normalized to chlorophyll content of wild type plants 14 days after germination. Error bars show standard deviation, ^∗^indicates significant differences to control tested by one-way ANOVA (*p* < 0.05).

Phytochrome B belongs to the family of plant photoreceptors that mediate physiological and developmental responses to light. The knockout *phyB-9* mutation was shown to affect hypocotyl elongation, chlorophyll content and flowering (Reed et al., 1993; Reed et al., 2000). This mutant was used to verify the utility of our image analysis system to reveal differences in rosette shape and size. In standard growth conditions rosette size of *phyB-9* was 40% smaller than wild type, due to elongated petioles and narrow leaves (**Figures 6A,B**). While Convex areas of the two genotypes were similar, Convex percentage of *phyB-9* was 40% smaller than Col-0, indicating differences in rosette shape (**Figures 6C,D**). Imaging revealed 10–15% lower chlorophyll content in *phyB-9* than in wild type plants (**Figure 6E**). These data demonstrate, that the PlantSize-based image analysis system is suitable to measure subtle differences in plant development, including shape and color.

### Technical Recommendations, Limitations of the Technology

While important parameters of young Arabidopsis plants could be efficiently quantified with the PlantSize application, the technology has certain limitations.

Imaging of closed Petri dishes can be problematic by condensation of water of the lid. Water condensation can often be avoided by appropriate culture conditions. If water condensation is problem in long-term experiments, imaging of the plates upside down can generate images for estimation of rosette sizes. In such arrangement color determination is not possible and root system might mask part of the leaves. According to our experience, the slightly reduced values in rosette sizes does not represent serious problem in Arabidopsis, which has thin roots. Moreover, similar alterations affect wild type and mutant, treated and control plants. Our experience showed, that removal of the lid for few seconds during photography is possible without risking contamination when short-term experiments are performed (up to 3 days).

Measurement of rosette sizes provides reliable results until individual plants and leaves are separated. Manual adjustment can be used to separate individual plants in some scenarios, which is however, a time-consuming process. In our conditions plant growth was therefore monitored for no more than 14 days, which was usually enough to generate reliable data to estimate growth rates of young plants (Col-0 ecotype was used in most experiments).

Estimation of chlorophyll and anthocyanin content through changes in HUE value/pixel is simple method, which calculates average HUE values of different pigments. Due to reverse linear correlation between chlorophyll and anthocyanine contents and HUE values (when plotted to pixel numbers of rosette sizes) with only a narrow overlap, a simple linear model could be used to estimate chlorophyll and anthocyanine contents.

Our system was optimized for 3000 × 4000 pixel image size, which should be taken into consideration during imaging. Different image sizes will require adjustments and further calibration of the PlantSize program.

## Discussion

The image analysis system was developed to facilitate the easy and fast evaluation of basic characters of plants, which can be precisely measured by non-destructive imaging without the need to invest into expensive hardware and software. Use of non-destructive methods in plant phenomics is increasingly attractive due to possibility to perform multiple measurements, acquire data on individual plants in multiple time points. Methods based on image analysis facilitate measuring of the observed parameters in time, calculation of kinetics, description of phenotypic and physiological plasticity, generation of timelaps presentations (Grosskinsky et al., 2015; Vanhaeren et al., 2015).

High capacity commercial phenotyping platforms produced by several companies such as LemnaTech^8^ or PSI^9^ offer automatic handling of large number of plants and imaging with multiple sensors, and complex image analysis. Several computer applications have been created for more simple purposes, to facilitate the analysis of particular morphological and physiological features (**Table 1**). While earlier softwares require manual acquisition of images, and analyze individual plants (Bylesjo et al., 2008; Schindelin et al., 2015), more recent applications are capable to perform simultaneous measurements of several plants, which is needed for high throughput analysis (De Vylder et al., 2012; Green et al., 2012; Minervini et al., 2017; Tome et al., 2017). Specific computer application have already been developed to estimate chlorophyll content on base of HUE or RGB values of color images, they typically do not give information on morphological features (Majer et al., 2010; Liang et al., 2017).

**Table 1:** Comparison of PlantSize with other imaging tools, developed for quantification of different plant parameters.

Name	Detection	Plant culture	Size	Shape	Color	Programing language, software	Output format	Reference
LAMINA	Individual	Soil	Yes	Yes	No	Java	txt	Bylesjo et al., 2008
HUE testing	Individual	Soil	No	No	Yes	MatLab	xls	Majer et al., 2010
ImageJ	Individual/Multiple	*In vitro*/soil	Yes	Yes	No	Java, R	txt	Schindelin et al., 2015
RosettR	Multiple	*In vitro*	Yes	Yes	No	R	R file	Tome et al., 2017
Color Checker	Multiple	*In vitro*	No	No	Yes	ImageJ Photoshop Matlab	csv	Liang et al., 2017
Phenotiki	Multiple	Soil	Yes	Yes	No	MatLab, CyVerse Cloud	csv	Minervini et al., 2017
Phenophyte	Multiple	Soil	Yes	Yes	No	Web-based, C language	csv	Green et al., 2012
Rosette Tracker	Multiple	Soil	Yes	Yes	No	JAVA ImageJ,	txt, csv, xls	De Vylder et al., 2012
PlantScreen Analyzer	Multiple	Soil	Yes	Yes	Yes			Awlia et al., 2016
PlantSize	Multiple	*In vitro*	Yes	Yes	Yes	MatLab	xls	This paper

The PlantSize application is able to perform simultaneous analysis of a number of plants (up to 36 plants were tested in the present version) and has the capability to simultaneously analyze size, shape and color of the plants. The PlantSize-based system therefore offers simultaneous analysis of the most commonly studied morphological parameters describing size and shape and provides information on chlorophyll and anthocyanin contents of the same plant. Our technology is rather simple and does not need heavy investment, as it relies on standard laboratory equipment, a digital camera and a standard desktop computer. The technology is therefore available for all research and biotechnology laboratories, which needs high throughput image analysis, but cannot afford an expensive phenotyping platform.

Using image analysis with PlantSize, differences in growth rates of wild type and transgenic plants could be revealed in control or saline conditions (**Figure 5**). Differences in plant shape and sensitivity to various stress conditions of different mutants could also be characterized (**Figure 6** and Supplementary Figure S6). Analysis of several morphological and physiological traits was shown to be important to reveal differences in responses to early or late phases of salt stress of different Arabidopsis genotypes (Awlia et al., 2016). That system, however, employs a complex phenotyping platform with several sensors and a complex software able to analyze simultaneously multiple data and traits.

To demonstrate the utility of our system, effect of heat shock factor A4A on plant growth was investigated by evaluating changes of rosette sizes, chlorophyll and anthocyanin accumulation of *HSFA4A* overexpressing plants or the *hsfa4a* knockout mutant in several stress conditions. Results confirmed earlier observations suggesting that *HSFA4A* can modulate responses to environmental stresses (Perez-Salamo et al., 2014). Capacity to evaluate differences in rosette shape and color was demonstrated by comparing rosette area, convex area and percentage and chlorophyll content of *phyB-9* mutants and wild type plants. Our method is suitable to reveal small but significant differences in plant sizes, shapes and color, which can contribute to the functional characterization of important regulatory genes such as the transcription factor HSFA4A or the light receptor phytochrome B.

The technology has been optimized for Arabidopsis. *In vitro* grown seedlings and small plants of other species can also be analyzed if images are generated with white background. Adaptation of the methods to other plants species, however, requires optimization of the experimental conditions and calibration of PlantSize. The PlantSize application is freely available and can be downloaded with documentation, which includes recommendations for installation and calibration of the software^10^. Potential users should check for updates of the software, and might consult with L, Sass for technical advice (sass.laszlo@brc.mta.hu).

## Author Contributions

DF, IV, and NA performed the experiments. LSa designed and created the PlantSize computer application. LSz designed the experiments, evaluated the data and wrote the manuscript. All authors read and approved the final manuscript.

## Conflict of Interest Statement

The authors declare that the research was conducted in the absence of any commercial or financial relationships that could be construed as a potential conflict of interest.

## Footnotes

1 http://www.plant-image-analysis.org/

2 http://www.lemnatec.com

3 http://www.psi.cz

4 https://www.wiwam.be

5 http://www.mathworks.com

6 http://www.brc.hu/pub/psize/index.html

7 https://imagej.nih.gov/ij/

8 http://www.lemnatec.com

9 http://www.psi.cz

10 http://www.brc.hu/pub/psize/index.html
