# Supplementary material for: PlantSize Offers an Affordable, Non-destructive Method to Measure Plant Size and Color in Vitro
Source: Front Plant Sci. 2018 Feb 22;9:219. doi: 10.3389/fpls.2018.00219 (PMC5827667; doi:10.3389/fpls.2018.00219)
Supplement: Supplementary file 1 [file Presentation_1.PDF]

The PlantSize application

Implementation

The PlantSize software was developed for multiple image analysis using MATLABs (version 2016b) with the Image Processing Toolbox™ (The MathWorks Inc., Natick, MA, USA, http://www.mathworks.com). The software is used to identify rosette objects and to calculate properties of those objects in a semi-automated fashion. The image analysis rapidly feasible on the user interface and requires minimal interaction. An example screenshot of the user interface is shown in Figure S2. Images which contains up to 36 plants were analyzed simultaneously. Data are exported in file format compatible with Excel. PlantSize generates data on rosette parameters such as Projected rosette area, Convex area, (Convex %), Weight and Colour appearance of plants: chlorophyll content (mg Chl./pixel), anthocyanine content (mg Anth./pixel). Convex % shows the ratio of area which is covered by green leaves within the convex area, when the total leaf area divided by the convex hull area. These calculations provide data about the shape of the leaves and rosette [23].

Description of image analysis

1. *Segmentation:* As inital step the Red-Green-Blue (RGB) colour space is converted to Hue, Saturation and Value color space (HSV). On a white background HSV parameters are used and object (plants) are defined with high accuracy and distinguished from the background using pixels which have higher saturation and lower value intensity. This colour thresholding step can be performed in a semi-automated way (ROI) and the user can set the sensitivity of the system. „Green" slider defines saturation value and „Grey" slider sets value intensity. The software recognize plants according to the green and grey settings. Define settings at the beginning of a set of measurement, and it will be applied automatically to each image. Post-process morphological step is adopted using `imerode` function. The above mentioned process can be determined according to the following series of formulas:

```
for i = 1 : numel(obj.sub_images)
   image_hsv = rgb2hsv(obj.sub_images(i).image);
   h = image_hsv(:,:,1);
   s = image_hsv(:,:,2);
   v = image_hsv(:,:,3);

   mask_s = s > obj.saturation_level;
   mask_v = v < obj.value_level;
   obj.sub_images(i).mask = mask_s & mask_v;
   obj.sub_images(i).mask =
   imerode(obj.sub_images(i).mask,strel('disk',2));
end
```

2. *Projected rosette area:* Plant size and weight analysis within the selected area of interest a modified Hue value was assigned, shifted colour space (-0.5 – 0.5) works well since the segmented object doesn't have any blue pixel. The area of each plant is estimated as sum of each pixel in the masked area and the weight is calculated from the amount of pixels.

```
obj.im_result = obj.image;
for i = 1 : numel(obj.sub_images)
   local_image = obj.sub_images(i).image;
   local_image_hsv = rgb2hsv(local_image);
   h = local_image_hsv(:,:,1);
   if ~isempty(obj.sub_images(i).user_mask)
      local_mask = obj.sub_images(i).mask &…
      obj.sub_images(i).user_mask;
      h(~local_mask) = 0;
```

```
            obj.sub_images(i).value = sum(local_mask(:));
            obj.sub_images(i).weight = 0.0005929 * obj.sub_images(i).value;
        else
            local_mask = obj.sub_images(i).mask;
            h(~local_mask) = 0;
            obj.sub_images(i).value = …
            sum(obj.sub_images(i).mask(:));
            obj.sub_images(i).weight = 0.0005929 * obj.sub_images(i).value;
        end
    end
```

3. Rosette colour analysis: After recording of rosette areas, chlorophyll and anthocyanine content are calculated from the correlation between Hue value and pixel number. During the estimation clarification for negative values and application of `histogram.BinEdges` and `histogram.BinCount` formulas is expedient to build in the script.

```
obj.sub_images(i).h_value = sum(h(:)) / obj.sub_images(i).value;
    obj.sub_images(i).chl_value = …
        (17.98 * obj.sub_images(i).h_value - 3.33) *…
        obj.sub_images(i).value / 1000.0 / 2.3815;
    if obj.sub_images(i).chl_value < 0
        obj.sub_images(i).chl_value = 0.0;
    end
    obj.sub_images(i).ant_value =…
        (-1.035 * obj.sub_images(i).h_value  + 0.2359) *…
        obj.sub_images(i).value;
    if obj.sub_images(i).ant_value < 0
        bj.sub_images(i).ant_value = 0.0;
    end

    h(~local_mask) = NaN;
    x_bins = -0.5:1/256:0.5;
    h_hist = histogram(h,x_bins);
    obj.sub_images(i).chl_value_pixel = 0;
    obj.sub_images(i).ant_value_pixel = 0;
    for i2 = 2 : numel(h_hist.BinEdges)
        value_pixel = h_hist.BinCounts(i2 - 1) * …
           (17.98 * h_hist.BinEdges(i2) - 3.33) / 1000.0 / 2.3815;
        if value_pixel > 0
            obj.sub_images(i).chl_value_pixel = …
                obj.sub_images(i).chl_value_pixel + value_pixel;
        end
        value_pixel = h_hist.BinCounts(i2 - 1)  * …
           (-1.035 * h_hist.BinEdges(i2)  + 0.2359);
        if value_pixel > 0
            obj.sub_images(i).ant_value_pixel = …
                obj.sub_images(i).ant_value_pixel + value_pixel;
        end
     end
```

4. Further shape parameters (convex hull area and percent):

```
    [obj.sub_images(i).convex_y,obj.sub_images(i).convex_x] = …
        find(local_mask);
    if isempty(obj.sub_images(i).convex_y)  || …
        isempty(obj.sub_images(i).convex_x)
        obj.sub_images(i).convex_hull = NaN;
        obj.sub_images(i).convex_area = NaN;
        obj.sub_images(i).convex_percent = NaN;
    else
```

```
    [obj.sub_images(i).convex_hull,obj.sub_images(i).convex_area] = …
       convhull(obj.sub_images(i).convex_x,obj.sub_images(i).convex_y);
    obj.sub_images(i).convex_percent = obj.sub_images(i).value / …
       obj.sub_images(i).convex_area;
end
```

Output format

Subsequent data processing can be performed in MS Excel. Saved data can be imported or copied into MS Excel or other statistical software. Standard statistical analysis can include calculation of averages, standard deviation, standard error, significance calculation, calculations of growth rates (Logest or Linest functions) and etc.

MATLAB Compiler

1. Prerequisites for Deployment

. Verify the MATLAB Runtime is installed and ensure you
  have installed version 9.1 (R2016b).

. If the MATLAB Runtime is not installed, do the following:
  (1) enter

      >>mcrinstaller

      at MATLAB prompt. The MCRINSTALLER command displays the
      location of the MATLAB Runtime installer.

  (2) run the MATLAB Runtime installer.

Or download the Windows 64-bit version of the MATLAB Runtime for R2016a
from the MathWorks Web site by navigating to

   http://www.mathworks.com/products/compiler/mcr/index.html

For more information about the MATLAB Runtime and the MATLAB Runtime installer, see
Package and Distribute in the MATLAB Compiler documentation
in the MathWorks Documentation Center.

NOTE: You will need administrator rights to run MCRInstaller.

2. Files to Deploy and Package

Files to package for Standalone
================================
-PlantSize.exe
-MCRInstaller.exe
   -if end users are unable to download the MATLAB Runtime using the above
    link, include it when building your component by clicking
    the "Runtime downloaded from web" link in the Deployment Tool
-This readme file

3. Definitions

For information on deployment terminology, go to
http://www.mathworks.com/help. Select MATLAB Compiler >
Getting Started > About Application Deployment >
Deployment Product Terms in the MathWorks Documentation
Center.
