# Supplementary material for: PlantSize Offers an Affordable, Non-destructive Method to Measure Plant Size and Color in Vitro
Source: Front Plant Sci. 2018 Feb 22;9:219. doi: 10.3389/fpls.2018.00219 (PMC5827667; doi:10.3389/fpls.2018.00219)
Supplement: Supplementary file 2 [file Presentation_2.PDF]

IMAGE ANALYSIS WITH PLANTSIZE

1. Introduction

Plant phenotype is determined by the genetic background and environmental conditions. Interaction of the genotype and environmental factors influences plant growth and development, physiological and molecular traits. Characterization of phenotypes is crucial to understand the regulation of stress responses on Arabidopsis model plant. Different useful software/plugin available to get data about phenomics parameters but all of them give data about one special trait on an expensive and time consuming way.

Here we get an inside view of high-throughput and affordable system, in which a Matlab based image analysis software is employed for reproducible analysis of several important growth parameters and color components. The following parameters are measured by the PlantSize otvare:

- **Projected rosette area (**Pixel Area): the area that is occupied by the green rosette in a top-view image in pixel unit.

- **The convex hull of the rosette** (Convex Area): The convex hull is the area defined by the smallest convex set containing the rosette in pixel unit.

- **Ratio of plant area within the convex hull** (Convex %): Ratio of the detected leaf area devided by the convex hull area in pixel unit,

- **Chlorophyll content:** The calculated chlorophyll content from measured Hue parameter of seedling in µg/pixel unit.

- **Anthocyanin content**: The estimated anthocyanin from Hue frequency of detected leaves in ng/pixel unit based on pixel and Hue color apparence parameters.

2. The supported phenotyping experiement

PlantSize can be used for phenomic characterization of *Arabidopsis thaliana* rosette leaves in vitro. Plants are grown in sterile condition on solid medium in plastic petri dishes and photographed at regular intervals. Images are then analysed with the program to estimate rosette parameter (area, weight), chlorophyll and anthocyanin content of the seedlings over time. These estimated values can then used to compare the differences between genotypes and the effect of applied treatments.

3. Plant growth and imaging

PlantSize is optimized to analyse size and color of small plants on white background. The system have been extensively tested with in-vitro grown Arabidopsis plants, but other seedlings and plantlets can also be analysed. Plants are optimally grown in square Petri dishes, arranged in a matrix. Identification and analysis of individual plants is easy in such arrangement.

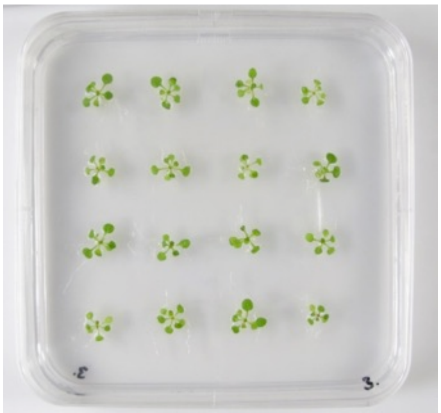

Figure 1. Arabidopsis plants grown in square Petri dish for PlantSize analysis.

Imaging can be done with any digital camera which can generate at least 3000x4000 pixel images, in RGB color. Images should be generated on white surface, with even illumination. Images should be saved in high quality JPEG files. We have used a Canon PowerShot SX20 digital camera with the following settings: ISO: 100, shutter speed: 1/30 sec, Aperture: F/5.6, Manual focus (20 cm), Exposure mode: macro. Conditions for digital photography should however be tested and optimized before large-scale experimets.

Important notes for imaging

- High contrast between rosette and background are necessary.
- White background with even illumination.
- Avoid reflection on the picture. It gives false value during the evaluation.
- Always use same resolution in an experiment. The actual version of PlantSize has been optimized for 3000x4000 pixel image size. Larger pictures should be adjusted to this image size.

4. Image analysis

Digital images in JPEG format should be copied into the hard disk of the computer in a separate folder.

Start the PlantSize program. Once the application is loaded, a welcome window appear. Click 'OK' button than two the Main and the Magnify windows will open. Most important steps of image analysis are described bellow.

4.1. Import an image file from the selected folder

File Menu > Load

Select for the image file, and import.

Other functions of the File menu:

File Menu > Save

Exports the most recent result of the designated folder in .xls format.

File Menu > Exit

Quit PlantSize.

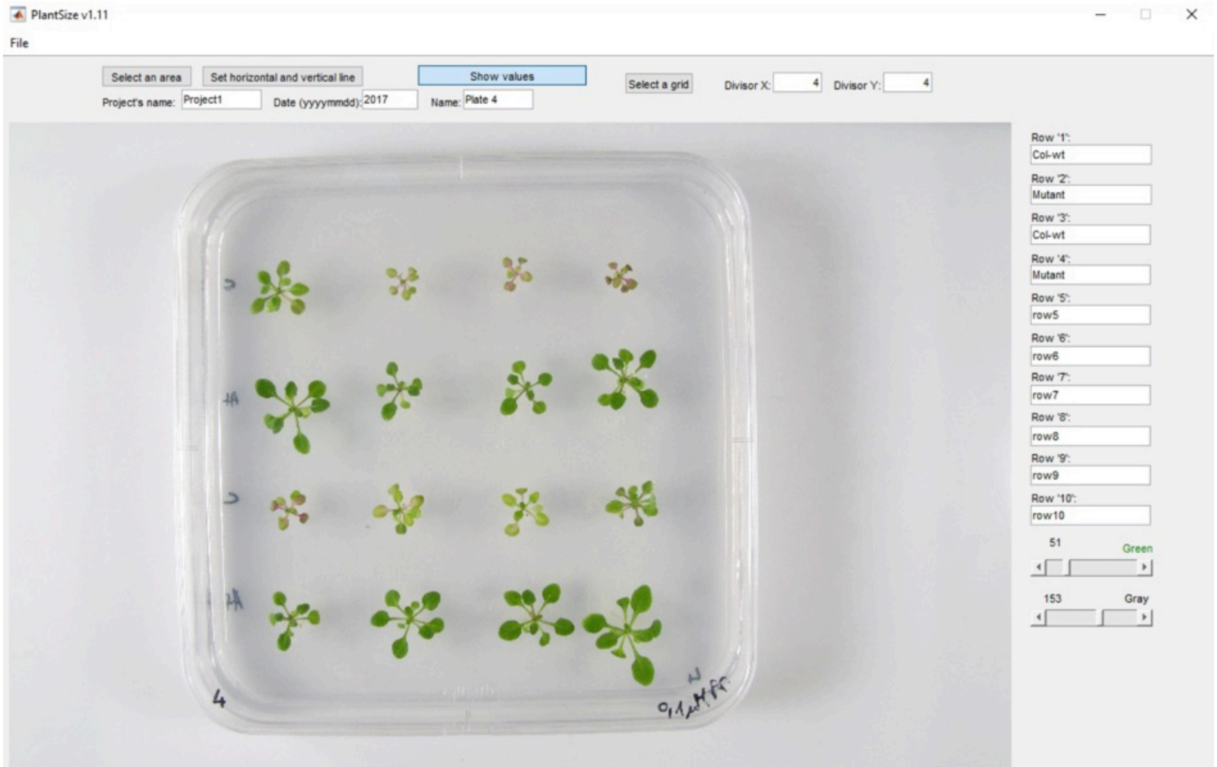

Figure 2. Dialog box of PlantSize with an imported image.

This particular square Petri dish has 16 plantlets, arranged in a 4x4 matrix, grown on medium which was supplemented with 0,1μM paraquat. First and third rows have wild type Col-0 plantlets, while in second and fourth rows paraquat tolerant S10 transgenic lines (Rigó et al,, 2016) were grown.

4.2. Set name, date (optional).

If you write a name in to the dialog box „project name”, than the exported excel file will have the same name. If the dialog box is left empty, than the exported file will have the name of the image.

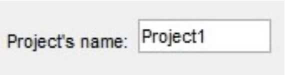

If you set the date, the same date will appear in exported excel sheet.

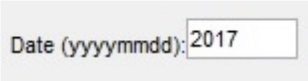

.

You can define a name of the analysis.

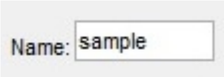

Define the name of the analysis (optional). It is the name of your Excel sheet in the exported file.

4.3. Define the matrix for analysis.

Enter the number of rows and columns into the dialog boxes Divisor X (column) and Divisor Y (row), which generates the matrix for analysis. For the example file, these numbers are the following.

Column:

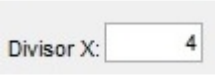

Row:

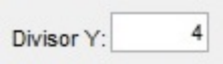

To define the matrix click the 'Select an area' button.

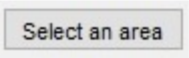

Click the upper left than the down right corner of the images of the plate. A blue matrix with even boxes will appear in the dialog box showing the positions of the areas to be analysed (Figure 3.). Individual boxes can be selected and enlarged for visual inspection of the images of the plants.

If necessary, matrix can be set manually by setting the horizontal and vertical lines with the following dialog box:

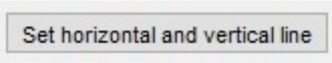

To set vertical divisions, click between each column to be separated. Number of vertical lines is the value of 'Divisor X' plus one.

To set horizontal divisions, click between each row, number of horizontal lines is the value of 'Divisor Y' plus one. The matrix is established by the defined web of vertical and horizontal lines.

4.4. Define the names of the rows.

Names of each row can be defined in the right side of the panel (Figure 3). In this version of PlantSize names of up to 10 rows (but not columns) can be defined. Therefore it is advisable to arrange different plant genotypes in separate rows. Names of each rows will appear on the header of rows in the exported excel file.

4.5. Enlarge selected images (optional).

In case you want to inspect an image in more detail, you can enlage its image in the 'Magnify' window by clicking the "Select a grid" box:

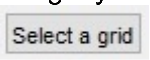

Select the plant of your interest, and click the selected cell. The selected plant will show up in the 'Magnify' window.

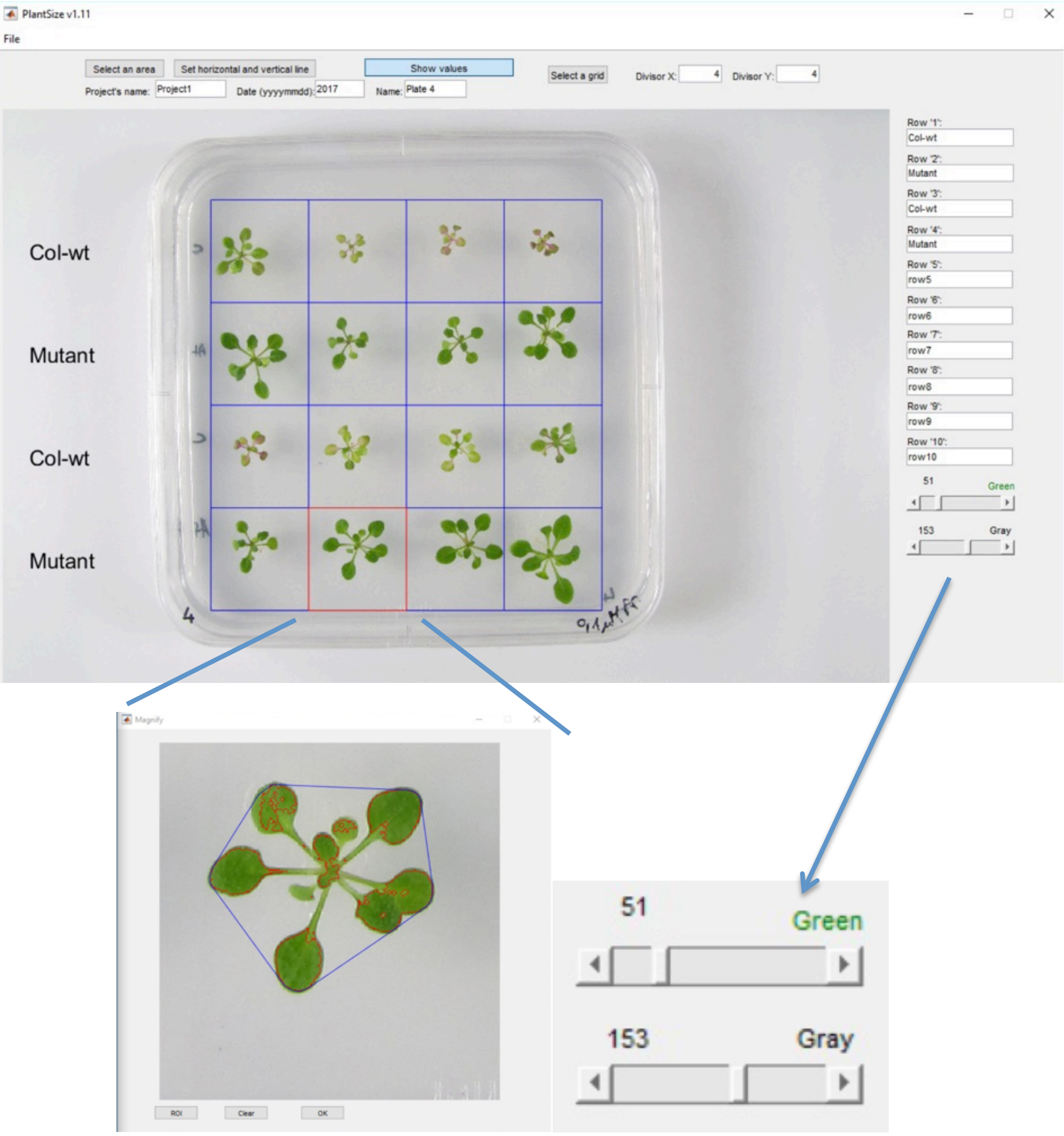

Figure 3. Dialog box of PlantSize with an imported image and defined matrix of 4x4 columns and rows. Emlarged image shows a typical arabidopsis plants with green rosettes. Sliders “Green” and “Gray” are in default position.

4.6. Segmentation

Using the “Green” and “Gray” sliders saturation and intensity of the images can be adjusted. In default state these sliders are in an intermediate position (Figure 3.). The default values are however rarely represent the optimal values for image analysis, which has to be set manually before analysis.

Slider “Green”

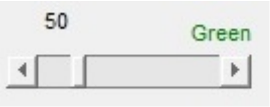

Slider Green sets the saturation value between 0-255 (in 8 bit). If you slider is decreased. the program will recognize and count more pixels. As PlantSize recognize leves and rosettes with their green color, changes in this parameter will modify the pixel number of selected rosette area (ROI).

Slider “Grey”

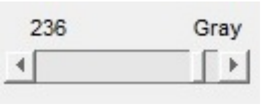

Using slider Gray, you can set the intensity of grayscale colour between 0-255 (in 8 bit). If you enhance Gray settings, than the program will recognize and count more gray pixels. This function helps you to select your plants. The changes of this parameter will influence the pixel number of the selected rosette area!

Green and Gray sliders have to be adjusted before a series of measurements to recognize and measure the largest and optimal areas of all plants (Figure 4.). The adjusted values of the sliders are applied for all images in consecutive measurements until they are modified or the PlantSize program is switched off.

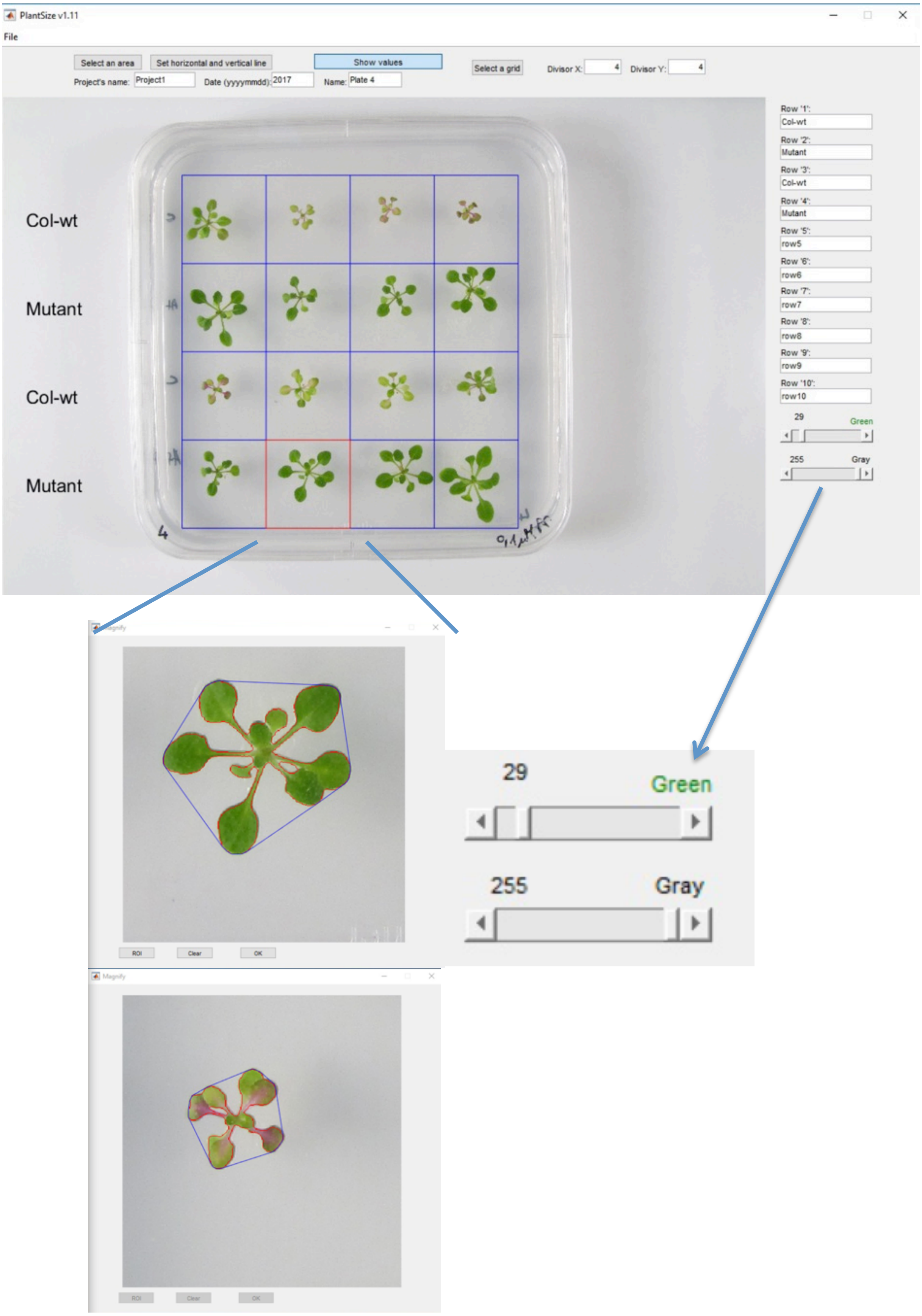

Figure 4. Dialogbox with adjusted Green and Gray sliders to optimize rosette recognition. Compare selected rosette areas of Figures 3. And 4. Note the differences in defined rosettes areas.

4.7. Show measured values (optional).

PlantSize offers the possibility to inspect numerical values of the measured characters on the scireen, If you click on the “Show values” dialog box, numerical values appear on each field of the matrix (Figure 5.). This is however not prerequisite for exporting and saving the data into an Excel-compatible file.

4.8. Export and save data.

PlantSize exports and saves data into an excel-compatible datafile (.xls format). Go to File Menu > Save. Data will be exported to the same folder with the image (Figure 5.).

If multiple images are analysed, do not close PlantSize, but open the next image file. Each dataset will be exported to the same file to different sheets with consecutive numbering.

4.9. Use of magnify window (optional).

You can enlarge the image of your plant of interest, To get a larger image, select the image with “Select a grid” button. This function is very useful when image analysis is adjusted with the “Green” and Gray” sliders (Figures 3, 4).

4.10. Use of ROI (Request Of Interest) function (optional)

With this function you can mask the enlarged cell image. If detected leaf area and convex hull are not match with the rosette of the chosen plant, a new polygone chain can be created manually.

1. Click ’ROI’ button.
2. Select a new polygon chain around the rosette with the mouse.
3. When new ROI line is completed, close the polygon chain with double click.

<u>Clear button</u>
When you click on this button the selected cell image will disappear and the previous calculation will be accessible.

<u>OK button</u>
With ’OK’ button you accept the adjusted mask and the phenotypic parameters will be calculated using the new mask.

Important notes!
Modifying either ’Green’ slider or ’Gray’ slider will clear all adjusted masks!

A

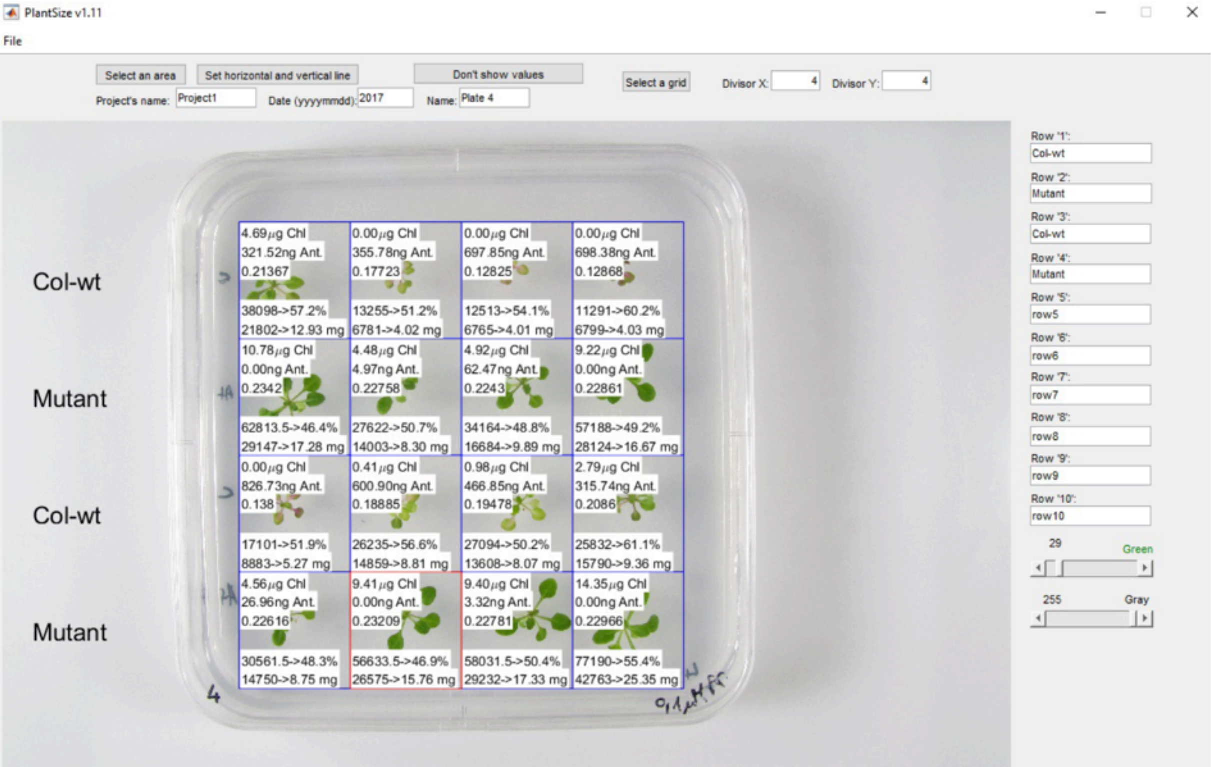

B

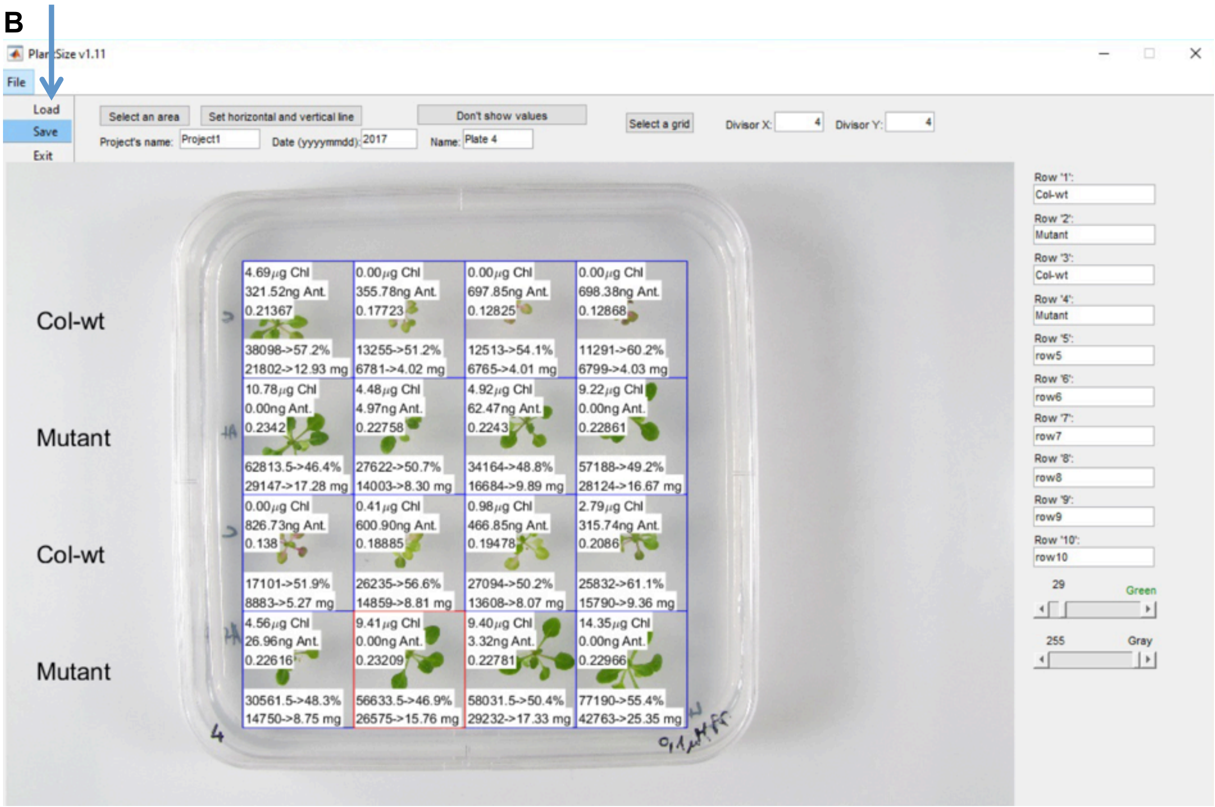

Figure 5. Display and save of numerical values. A) Numerical values of individual plants are displayed when „Show values" dialog box is used. Display can be reversed by clicking the „Don't show values" box. B) Data are exported and saved by clicking „Save" box in the „File" menu (arrow).

5. Software availability

The PlantSize software is freely available on the following site:
http://www.brc.hu/pub/psize/index.html.

For more details please contact with the developer:

Dr. László Sass:

E-mail: lsass@brc.hu
Web: http://www.brc.hu/personal_page.php?id=nb_sal%C3%A1
