# Supplementary figures and images for: PlantSize Offers an Affordable, Non-destructive Method to Measure Plant Size and Color in Vitro
Source: Front Plant Sci. 2018 Feb 22;9:219. doi: 10.3389/fpls.2018.00219 (PMC5827667; doi:10.3389/fpls.2018.00219)

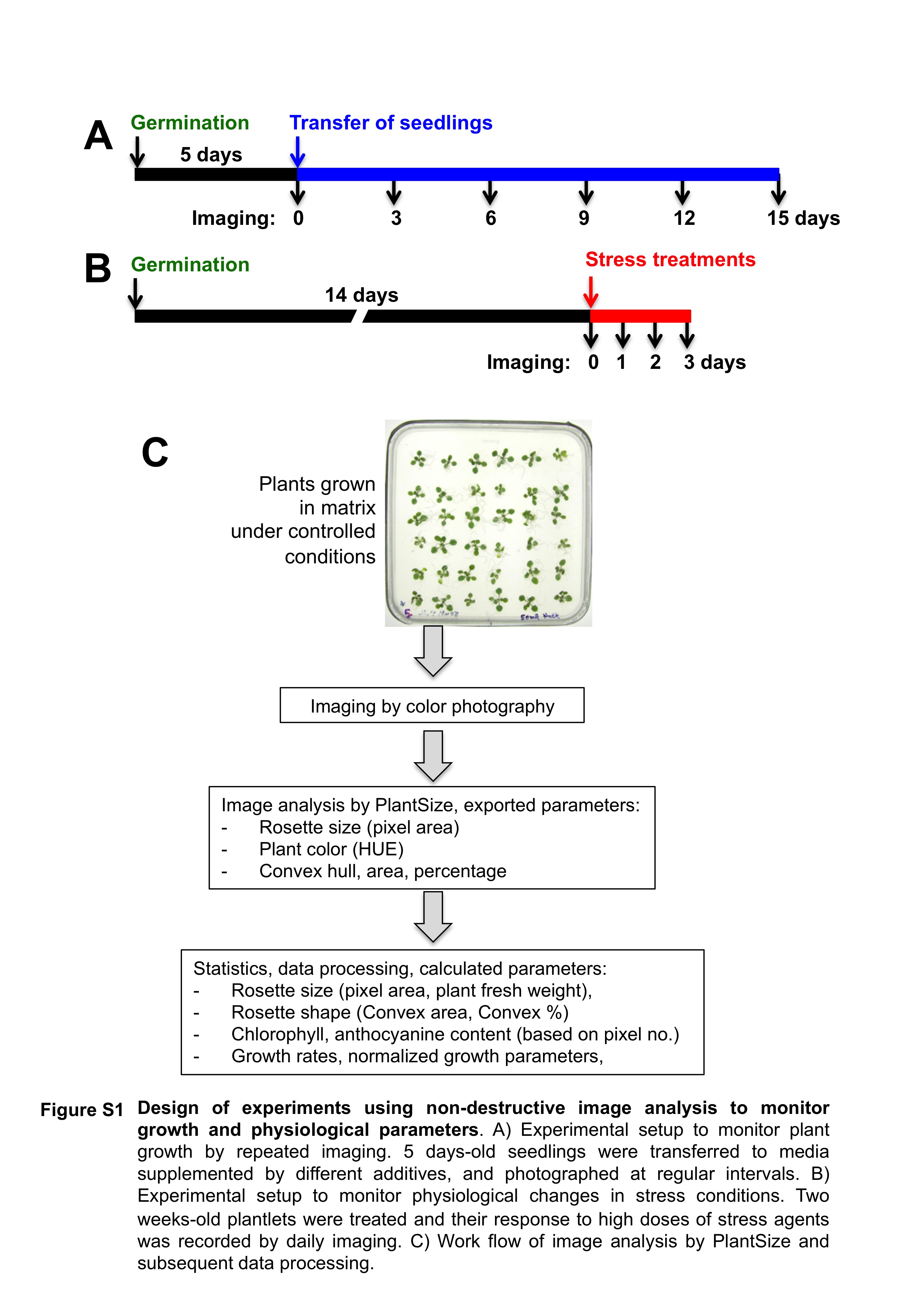

Supplement: Supplementary file 3 [file Image_1.JPEG]

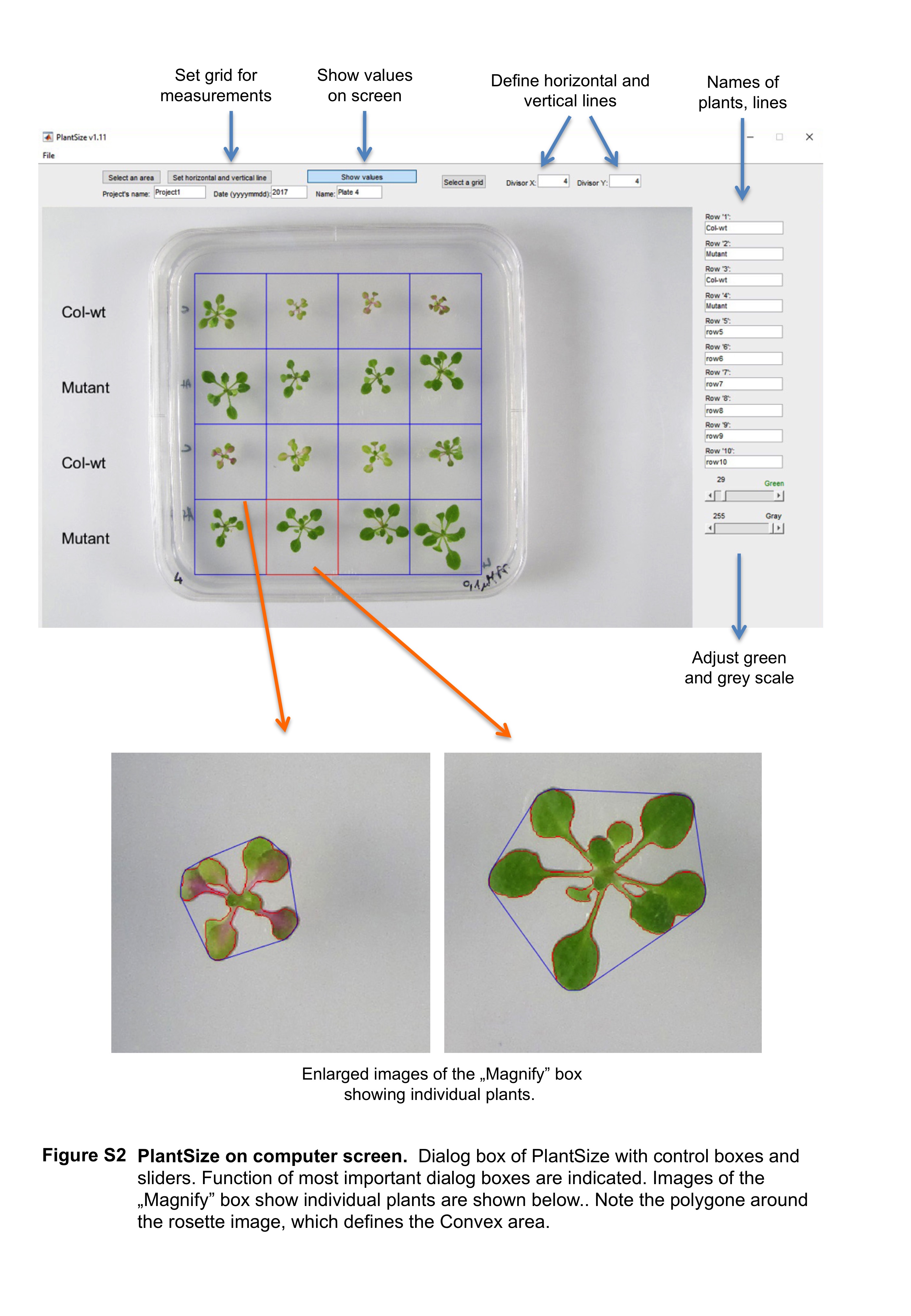

Supplement: Supplementary file 4 [file Image_2.JPEG]

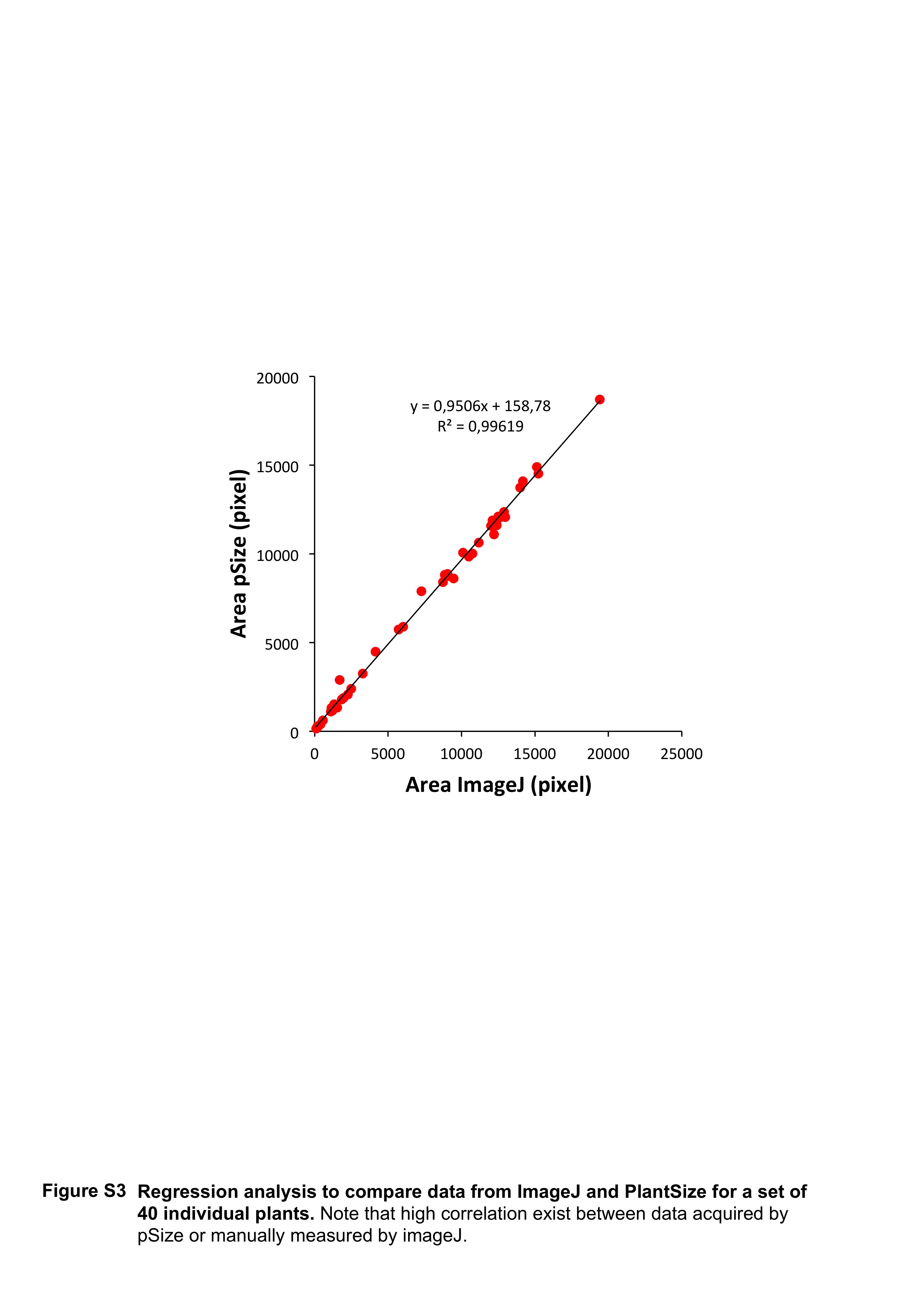

Supplement: Supplementary file 5 [file Image_3.JPEG]

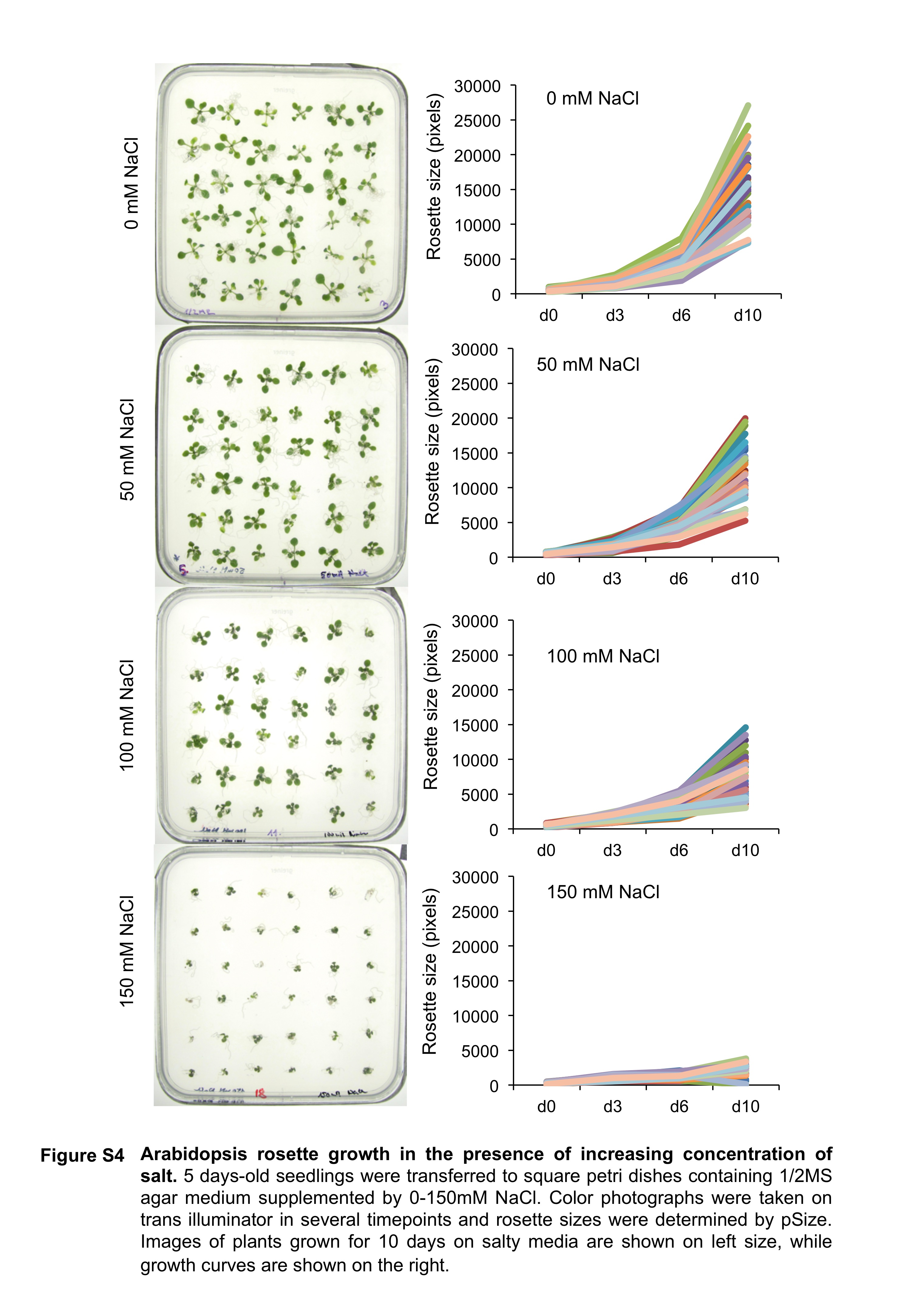

Supplement: Supplementary file 6 [file Image_4.JPEG]

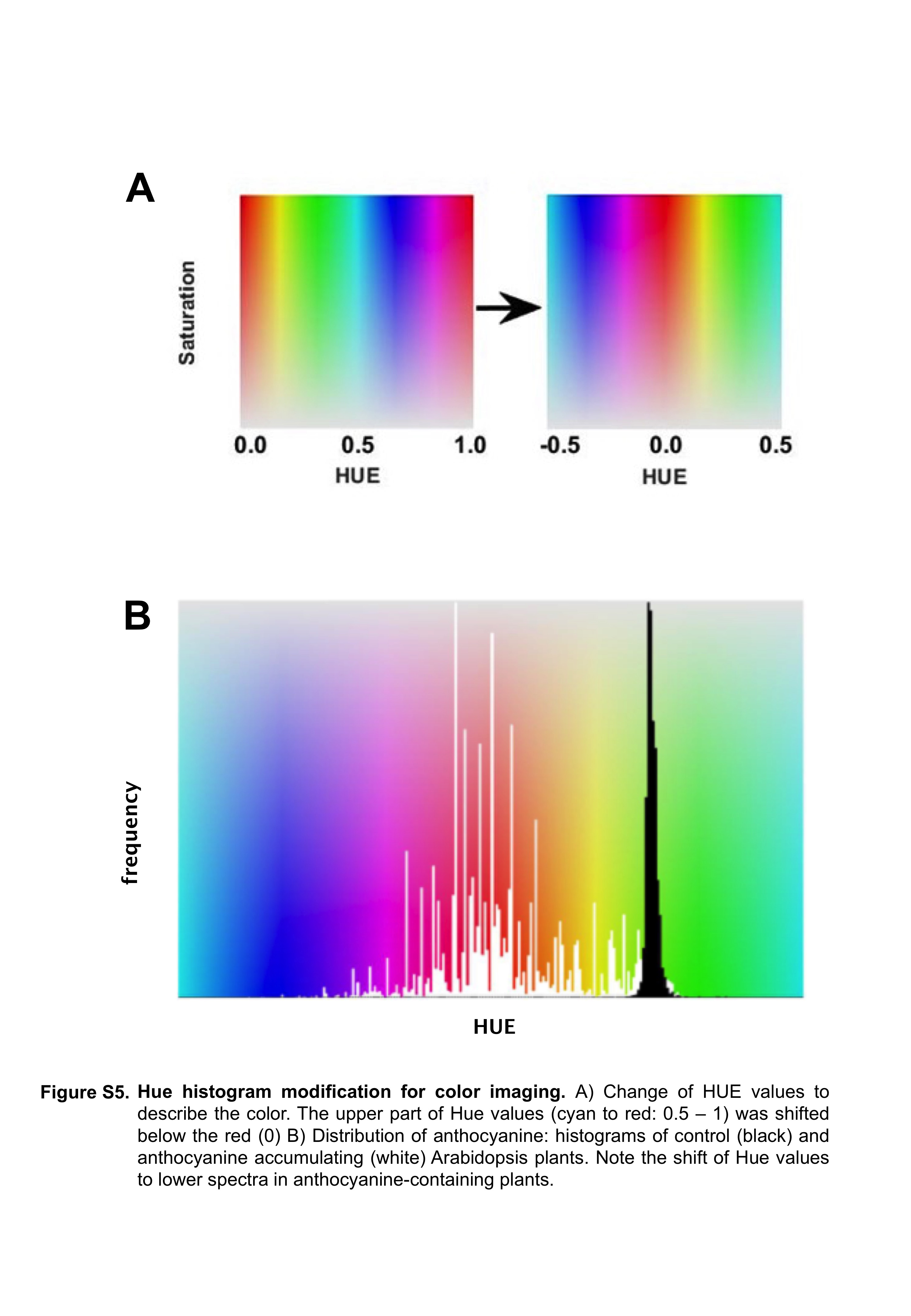

Supplement: Supplementary file 7 [file Image_5.JPEG]

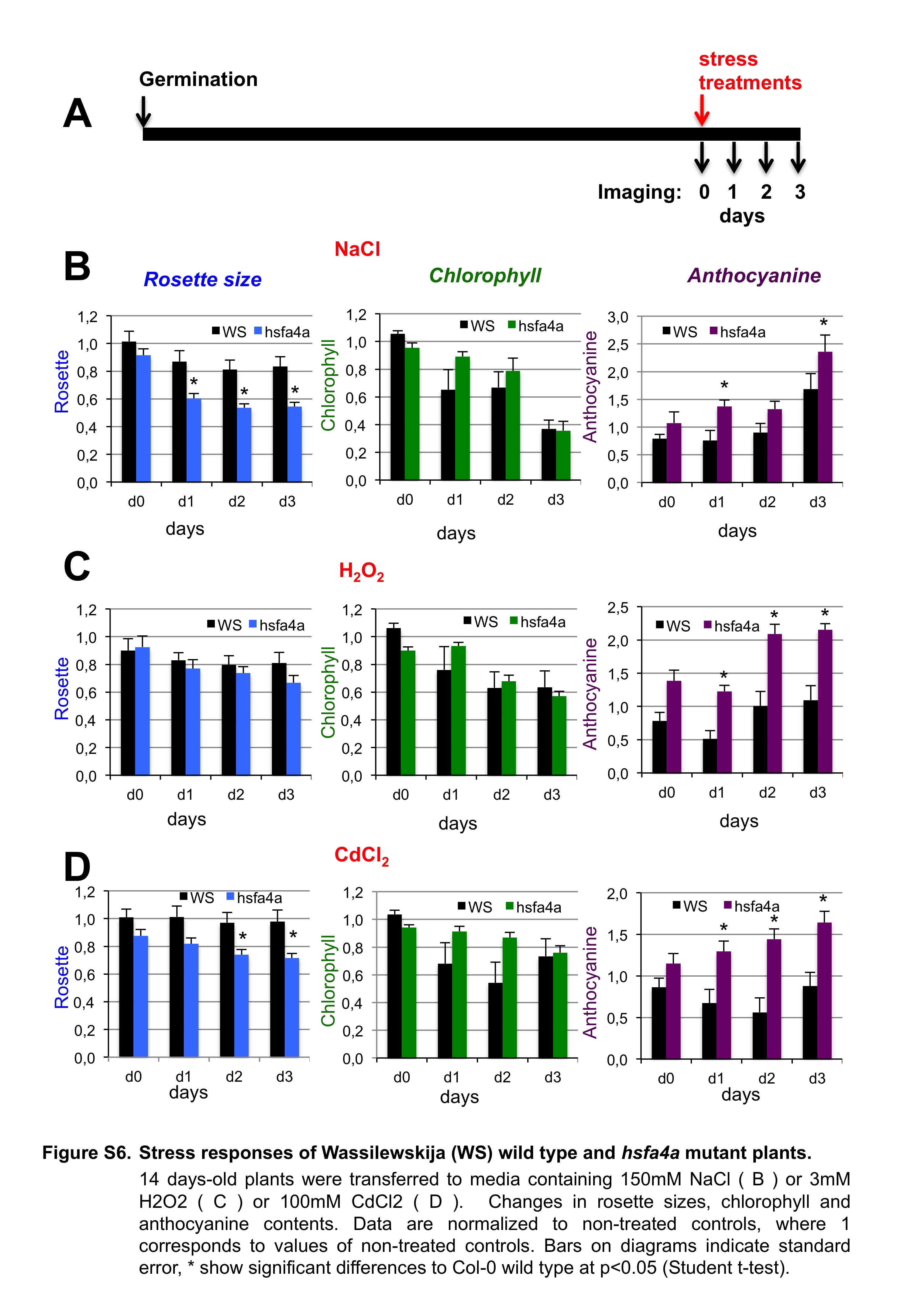

Supplement: Supplementary file 8 [file Image_6.JPEG]
